# Triple-acting Lytic Enzyme Treatment of Drug-Resistant and Intracellular *Staphylococcus aureus*

**DOI:** 10.1038/srep25063

**Published:** 2016-04-28

**Authors:** Stephen C. Becker, Dwayne R. Roach, Vinita S. Chauhan, Yang Shen, Juli Foster-Frey, Anne M. Powell, Gary Bauchan, Richard A. Lease, Homan Mohammadi, William J. Harty, Chad Simmons, Mathias Schmelcher, Mary Camp, Shengli Dong, John R. Baker, Tamsin R. Sheen, Kelly S. Doran, David G. Pritchard, Raul A. Almeida, Daniel C. Nelson, Ian Marriott, Jean C. Lee, David M. Donovan

**Affiliations:** 1 ARS, USDA, 10300 Baltimore Ave, Beltsville, MD, USA; 2 Biology, Univ. North Carolina, Charlotte, Charlotte, NC, USA; 3 Institute for Bioscience and Biotechnology Research, University of MD, Rockville, MD, USA; 4 Department of Veterinary Medicine, University of MD, College Park, MD, USA; 5 Channing Laboratory, Department of Medicine, Brigham and Women’s Hospital and Harvard Medical School, Boston, MA, USA; 6 Biochemistry, Univ. Alabama, Birmingham, Birmingham, AL, USA; 7 Biology, San Diego State University, San Diego, CA, USA; 8 University of Tennessee, Knoxville, TN, USA; 9 Dept. of Chemical & Biomolecular Engineering, The Ohio State University, Columbus, OH, USA.; 10 Institute of Food, Nutrition and Health ETH, Zurich, Zürich, Switzerland.; 11 Department of Biochemistry and Molecular Biology, Louisiana State University Health Sciences Center, New Orleans, LA, USA.; 12 Unité de Biologie Moléculaire du Gène chez les Extrêmophiles Département de Microbiologie, Institut Pasteur, Paris, France.

## Abstract

Multi-drug resistant bacteria are a persistent problem in modern health care, food safety and animal health. There is a need for new antimicrobials to replace over used conventional antibiotics. Here we describe engineered triple-acting staphylolytic peptidoglycan hydrolases wherein three unique antimicrobial activities from two parental proteins are combined into a single fusion protein. This effectively reduces the incidence of resistant strain development. The fusion protein reduced colonization by *Staphylococcus aureus* in a rat nasal colonization model, surpassing the efficacy of either parental protein. Modification of a triple-acting lytic construct with a protein transduction domain significantly enhanced both biofilm eradication and the ability to kill intracellular *S. aureus* as demonstrated in cultured mammary epithelial cells and in a mouse model of staphylococcal mastitis. Interestingly, the protein transduction domain was not necessary for reducing the intracellular pathogens in cultured osteoblasts or in two mouse models of osteomyelitis, highlighting the vagaries of exactly how protein transduction domains facilitate protein uptake. Bacterial cell wall degrading enzyme antimicrobials can be engineered to enhance their value as potent therapeutics.

*Staphylococcus aureus* is an opportunistic bacterial pathogen responsible for a diverse spectrum of diseases including mastitis, osteomyelitis, and endocarditis^1^. *S. aureus* rapidly develops resistance to antibiotics, as illustrated by multi-drug resistant (MDR), methicillin-resistant *S. aureus* (MRSA) and the reduced susceptibility to vancomycin (vancomycin-intermediate strains)^2^. The number of hospitalizations due to MRSA infection included $9.7 billion increased health care costs in 2005^3^,^4^.

*S. aureus*, including MRSA, can asymptomatically colonize the anterior nares of healthy humans, and from these reservoirs opportunistically infect the host. During an infection *S. aureus* has also been reported to reside intracellularly in certain host cells, *e.g.*, human nasal epithelial cells^5^, bovine mammary cells^6^,^7^, and avian osteoblasts^8^. Intracellular bacteria evade most antibiotics and the host immune system, allowing them to re-emerge after treatment and infect surrounding cells and tissues^9^. For example, *S. aureus* was the most common infective agent associated with reoccurring osteomyelitis (53%) in war zone blast wounds, with 60% of these strains being antibiotic-resistant^10^. A recent publication demonstrates *in vivo* efficacy with an antibody-antibiotic conjugate to eradicate intracellular staphylococci, however there are known resistance mechanism to most antibiotics^11^. There is a need for antimicrobials that are refractory to resistance development and can kill intracellular MDR bacteria.

Peptidoglycan hydrolases (PGHs) are candidate antimicrobials with properties that are ideal for treatment of MDR infections^12^. PGHs digest the bacterial cell wall peptidoglycan (PG), causing osmolysis and death^13^. The Gram-positive PG structure is highly divergent^14^, and PGH domains which target unique bonds in the PG lyse target bacteria often with near-species or serovar-specificity^15^. This high level of specificity avoids the adverse effects of antibiotic selection on unrelated commensal strains. PGHs have been shown to be versatile in their applications (for reviews see^16^,^17^,^18^).

Many PGHs are modular in structure with enzymatic and cell wall binding domains (CBDs) separated by flexible linkers^16^, allowing for recombinant manipulation and generation of chimeric molecules^19^,^20^,^21^. On the premise that a bacterium is unlikely to evade three simultaneous PGH activities, we engineered fusion proteins with three unique lytic activities and determined the impact of these chimeras on resistance development. We further modified these triple-acting fusion PGHs with protein transduction domains (PTDs) to facilitate entry into mammalian cells^22^, and demonstrated their ability to enhance the eradication of biofilm and intracellular staphylococci in multiple *ex vivo* and *in vivo* models.

## Results

### Engineering triple-acting fusion PGHs

**Figure 1:**
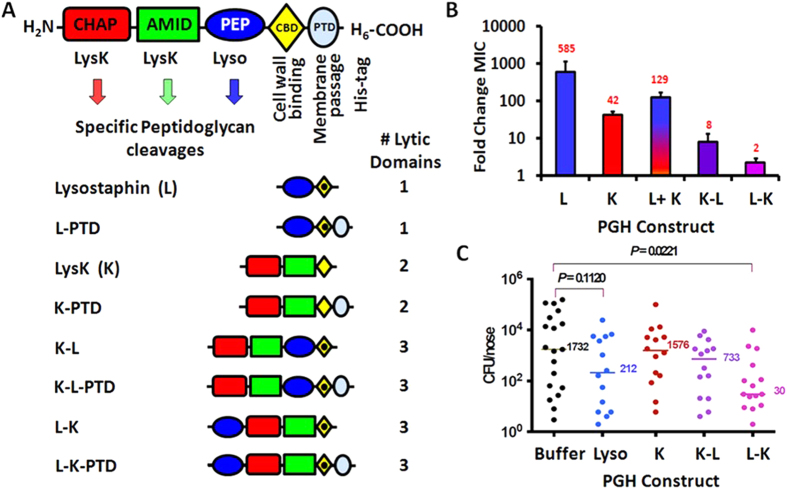
PGH construct schematics, resistance development and eradicating *S. aureus* in a rat colonization model. (**A**) Schematic of PGH constructs. Domains: CHAP endopeptidase (CHAP, red box); N-acetylmuramoyl-L-alanine amidase (AMID, green box); M23 endopeptidase (PEP, blue oval); CBDs (LysK SH3b, gold diamond; lysostaphin SH3b, gold diamond with dot); protein transduction domain (PTD, blue circle); hexahistidine purification tag (His_6_). Domains not to scale. Specific PG cut sites are illustrated in Supplementary Fig. 2B. B. *In vitro* antimicrobial resistance development. Engineered triple fusions K-L and L-K suppress antimicrobial resistance development compared to LysK (K), lysostaphin (L), or a combination of equimolar concentrations of both (L+K). Changes in MIC are depicted as a fold-change at the tenth round of sublethal exposure compared to the first exposure, with the average fold-change of 4 replicates in red. Error bars = SEM. First exposure MICs: Lysostaphin, 0.77 μg/ml (27 nM); LysK, 47 μg/ml (840 nM); Lysostaphin and LysK (L+K) in combination 0.2 μg/ml (7 nM and 3 nM respectively) ;triple fusion K-L, 7 μg/ml (97 nM); triple fusion L-K, 7.8 μg/ml (107 nM). C. Colonization reduction in a rat nasal carriage model. Rats were inoculated with *S. aureus strain* ALR on day 1. After 5 days, the rats were treated twice daily for 3 days with 20 μl of a 10 mg/ml solution of each enzyme. The rat noses were excised on day 10, homogenized, and quantitative cultures were performed. Each point represents the CFU recovered from an individual rat. Bars indicate the median CFU/nose recovered from treated rats. Triple fusion L-K showed a significant reduction in colonization (98%) of treated rats compared with rats treated with buffer alone. Data were compiled from five independent experiments. Lyso = commercially purchased lysostaphin (AMBI, Tarrytown, NY). Statistical comparisons were made with the Mann-Whitney test.

To impede resistance development, we engineered PGH fusion proteins to harbor three unique lytic activities from two parental PGHs (Fig. 1A). Lysostaphin^23^ contains a glycyl-glycine M23 endopeptidase domain, and LysK^24^ contains two catalytic domains, an N-terminal cysteine, histidine-dependent amidohydrolase/peptidase (CHAP endopeptidase) and an N-acetylmuramoyl-L-alanine amidase^25^. Both parental enzymes contain a C-terminal SH3b CBD. LysK and lysostaphin are known to be inactive against Gram negative bacteria, but show strong lytic activity against antibiotic-sensitive and antibiotic-resistant *S. aureus* and Coagulase negative strains (Supplementary Table 1), and in combination demonstrate synergy in killing *S. aureus* cells^13^. Each enzymatic domain targets separate unique bonds in the *S. aureus* PG^12^ (illustrated in Supplementary Fig. 1), making them ideal candidates for inclusion in our triple-acting fusions.

We previously described a head-to-tail fusion of LysK-lysostaphin that included two CBDs and showed weak staphylolytic activity^12^. Improvements were made to create two triple-acting constructs, each with a single CBD (the SH3b domain from lysostaphin). The first triple-acting PGH (K-L) was constructed with both LysK lytic domains fused to the N-terminus of lysostaphin. The second triple-acting construct (L-K) inserted the LysK lytic domains between lysostaphin’s M23 endopeptidase domain and SH3b CBD (Fig. 1A). Parental and triple-acting fusion proteins were expressed in *E. coli* and purified by nickel chromatography via a C-terminal His_6_ tag (~98% pure by SDS PAGE), yielding a single band in zymogram analysis (Supplementary Fig. 2A,B). The triple-acting PGHs demonstrated intermediate activity compared to the parental PGHs against Coagulase negative strains as well as antibiotic-sensitive and antibiotic-resistant *S. aureus* in both plate lysis (Supplementary Fig. 2C), minimum inhibitory concentration (MIC) assays (Supplementary Table 1), and were as effective as the parental PGHs in turbidity reduction assays (Supplementary Fig. 2D), which has been shown previously to equate to reduced bacterial viability^16^. Importantly, all three lytic domains were active in each of the fusion constructs examined, as illustrated in the representative electron spray ionization mass spectrometry results of PG digestion products created by K-L (Supplementary Fig. 1). The fusions were also as effective as the parental enzymes at eradicating static biofilms. Increased biofilm eradication was obtained at the highest concentrations tested when the parental enzymes were added together (Supplementary Fig. 3).

### Resistance development to triple-acting fusion enzymes

Development of resistance to the triple-acting fusion enzymes was tested *in vitro* on *S. aureus* strain Newman and compared to the parental enzymes applied individually or simultaneously. After 10 rounds of sub-lethal exposure in liquid culture, parental LysK (two lytic domains) and lysostaphin (one lytic domain) yielded cultures with 42-fold and 585-fold increases in MICs, respectively. When *S. aureus* cells were simultaneously exposed to equimolar mixtures of LysK and lysostaphin, the final MIC increased 129-fold (Fig. 1B). In contrast, K-L and L-K triple-acting fusion enzymes yielded cultures with a mere 8-fold and 2-fold increase in MIC, respectively (Fig. 1B, initial MICs are indicated in Supplementary Table 1). Survivors of the 10-round exposure to PGH were passaged five times in media with no PGH added. These passaged bacteria retained their elevated MIC, suggesting that resistance was not physiologically induced (e.g. selective conditioning), but was likely a result of genetic alterations. We compared our triple-acting fusions to small molecule antibiotics in similar assays. Trimethoprim/Sulfamethoxazole (1/20 ratio) and streptomycin were both assayed after 10 rounds of sublethal exposure and yielded 6-fold and 13-fold MIC increases, respectively. Resistance development during 10 rounds of sub-lethal exposure in plate lysis assays showed an 8-fold increase in resistance to lysostaphin, a 2-fold increase for LysK, and no detectable increase in resistance for triple fusions K-L or L-K.

### Triple fusion L-K reduces nasal carriage of *S. aureus*

To determine whether our triple-acting fusion enzymes showed *in vivo* potency, we tested the parental and chimeric PGHs in a robust nasal colonization model. Rats were inoculated on day 0, treated twice daily on days 3, 4 and 5, and euthanized for quantitative cultures of their nasal tissue on day 10. Treatment with triple-acting fusion L-K resulted in a 98% decrease in the colony forming units (CFU)/nose compared to rats treated with buffer (Fig. 1C). In contrast, equimolar concentrations of recombinant LysK or triple-acting fusion K-L were unable to significantly reduce the bacterial load. Rats treated with 200 μg lysostaphin (AMBI, Tarrytown, NY) showed an 87% (*p* = 0.085) reduction in nasal colonization, consistent with previous findings^26^. Colonies (N = 22) recovered from the nares of rats treated with the triple-acting fusion L-K were not more resistant than the parental strain in plate lysis analysis (not shown). The MIC of 8 post-treatment isolates toward triple fusion L-K was 7.0 ± 2.4 μg/ml (N = 32), similar to that of *S. aureus* ALR (9.4 ± 6.1 μg/ml (N = 8)). A separate experiment in which rats were treated twice per day with mupirocin, the currently available pharmaceutical “gold standard” for human nasal decolonization (scaled down in volume for a rat nose; 10 μl of 2% mupirocin) resulted in a 98% reduction in nasal carriage (Supplementary Fig. 4).

### PGHs linked to a PTD enhance eradication of intracellular *S. aureus*

**Figure 2:**
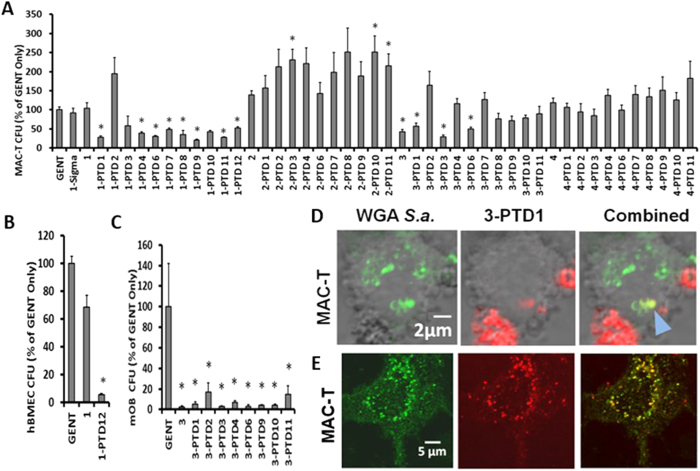
PGH-PTD eradication of intracellular *S. aureus* in cultured cells. Cultured cells were infected, treated with gentamicin to kill extracellular *S. aureus*, and then treated with the PGHs indicated. All results are standardized to the gentamicin (GENT) only control. Nomenclature of the PGH constructs is as in Fig. 1. PTDs are listed in Table 1. Asterisks indicate statistical significance detected with single factor ANOVA (α = 0.05) with paired t-test posthoc analyses α = 0.05 adjusted with Šidák correction for multiple comparisons (for all cases *p* < 0.01). (**A**) Bovine mammary epithelial cell line (MAC-T) infected with strain Newbould 305 (N = 8). Error bars represent SEM. 1-Sigma is commercial lysostaphin (Sigma). (**B**) Human brain microvasculature epithelial cells (hBMEC) infected with *S. aureus* strain ISP479C (N = 3). (**C**) Murine primary osteoblasts (mOB) infected with *S. aureus* strain UAMS-1 (N = 3). (**D**) Single plane of confocal microscopy z-stack overlaid on bright field exposure of live cultured MAC-T cells exposed to both *S. aureus* and PGH 3-PTD1. Live *S. aureus* (~0.6–1.0 μM diameter) are labeled with green fluorescent *wheat germ* agglutinin (WGA); PGH K-L-PTD1 is labeled with Alexa Fluor (Red). Yellow staining in the combined panel represents intracellular co-localization (in a single plane as determined by z-stack analysis) of both *S. aureus* and K-L-PTD1 (blue arrow). (**E**) Confocal microscopy maximum intensity projections (with all z-planes represented) of a MAC-T cell exposed to *S. aureus* and K-L-PTD1 as in **(D**) The majority of the *S. aureus* are localized in the thickest part of the cytoplasm surrounding the zone of exclusion created by the nucleus. Many, but not all, *S. aureus* are co-localized with K-L-PTD1 (yellow) in the combined panel.

PTDs are short highly cationic peptide sequences of ~9–30 amino acids that occur naturally and facilitate protein transduction across eukaryotic cell membranes^27^. Parental enzymes (lysostaphin, LysK) and triple-acting fusions K-L and L-K were each modified by addition of 11 different C-terminal PTD sequences (Table 1; schematic Fig. 1A) and were tested for their ability to reduce intracellular *S. aureus* in multiple cultured cells known to support *S. aureus* intracellular invasion (Fig. 2A,B and C). Neither commercial lysostaphin (1-Sigma; Sigma-Aldrich, St. Louis, MO) nor the C-terminal His_6_-tagged lysostaphin were able to decrease the intracellular CFUs of *S. aureus* in a cultured bovine mammary epithelial cell line (MAC-T) or cultured human brain microvasculature endothelial cells (hBMEC) (Fig. 2A,B). However, the addition of PTDs to the C-terminus of His_6_-tagged lysostaphin resulted in significant reductions in intracellular *S. aureus* in both cell lines. In MAC-T cells, virtually all tested constructs displayed this effect, despite the fact that modification with PTDs often reduced the enzymatic activity of most constructs tested (Supplementary Fig. 5). A striking example was the ability of construct L-PTD12 to reduce the intracellular *S. aureus* strain ISP479C^28^ from hBMEC cells in culture (Fig. 2B). In contrast, the ability of LysK or L-K fusion to eradicate intracellular *S. aureus* in MAC-T cells was inhibited by the addition of a PTD (Fig. 2A). Interestingly, triple-acting fusion K-L reduced the intracellular bacteria recovered from either MAC-T cells or murine osteoblasts (mOB), and this effect was not significantly enhanced with the addition of a PTD (Fig. 2A,C).

To confirm protein transfer across mammary epithelial cell membranes, cultured MAC-T cells were exposed to fluorescently labeled K-L-PTD1 (red) and fluorescently labeled *S. aureus* strain Newman (green) and monitored in real-time with confocal microscopy. *S. aureus* strain Newman and the triple fusion K-L-PTD1 were found to co-localize intracellularly in a single z-plane within MAC-T cells (Fig. 2D). A similar result was apparent in the maximum intensity projections (with all z-planes visualized) (Fig. 2E). To ensure that neither the exposure to PGH-PTD fusions nor the combination of the PGH-PTD fusions and *S. aureus* was cytotoxic in these *ex vivo* assays, trypan blue staining^29^ was performed on MAC-T cells that were exposed to either *S. aureus* or *S. aureus* and selected PGH-PTD fusions for two hours [per the exact protocol used to measure intracellular eradication of *S. aureus*]. No cytotoxic effects were observed with only 2–5% of the intact monolayer staining blue with either buffer alone or any of these high activity constructs: Lyso, Lyso-PTD1, Lyso-PTD9, K-L, K-L-PTD1, and K-L-PTD9.

**Figure 3:**
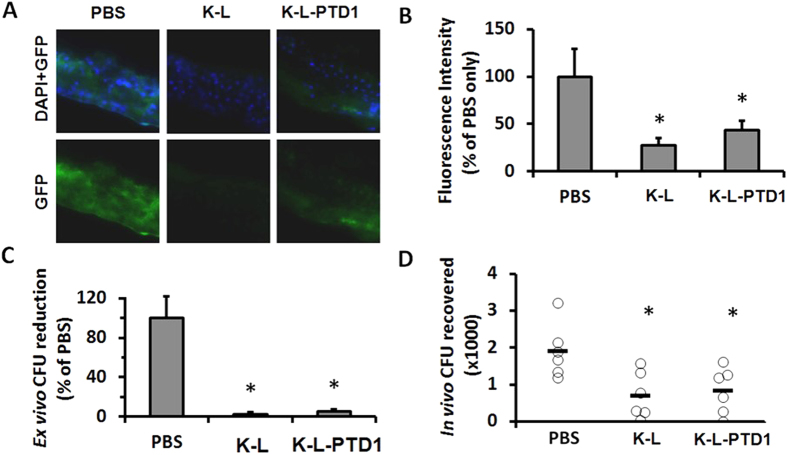
Triple-acting fusions can eradicate intracellular *S. aureus* in murine osteoblasts. (**A**) Intracellular eradication of GFP expressing *S. aureus.* Neonatal mouse whole calvaria were treated for 4 hours with chimeric PGHs or buffer alone, 24 h post inoculation with GFP expressing *S. aureus.* Calvaria were embedded in freeze media, sectioned, and subjected to fluorescence imaging. The image shown is a representative figure for triplicate sections of three separate calvaria. (**B**) Fluorescence intensity from these sections was measured in ImageJ and defined as arbitrary fluorescence standardized to the untreated control. Error bars represent SEM of four replicate experiments. (**C**) *Ex-vivo* intracellular *S. aureus* eradication. Infected calvaria were homogenized and CFU were counted post treatment. Error bars represent SEM. (**D**) Murine model of staphylococcal osteomyelitis. C57BL/6J mice were anesthetized and their femurs were surgically exposed. A trough was drilled through the bone cortex, and the damaged bone sites were inoculated with 1 × 10^3^ CFU *S. aureus* in agarose beads. After 24 hours, mice were treated (i.m. to site of infection) twice in a 24 h period with PBS or 5 mg/kg of triple fusion K-L or K-L-PTD1. The femurs were removed, homogenized, and plated to quantify the bacterial load. Bars indicate the average CFU recovered (N = 6). Both triple fusion K-L (*p* = 0.012) and K-L-PTD1 (*p* = 0.021) significantly reduced bacterial load as compared to no treatment, but the presence or absence of PTD1 had no significant effect (*p* = 0.73). Asterisks represent statistical significance as determined by one-way ANOVA followed by Tukey’s posthoc test.

To examine intracellular uptake and eradication in murine osteoblast containing tissue samples, both an *ex vivo* calvaria (skull cap) and *in vivo* femur injury model were tested. Both the triple-acting fusion K-L and a PTD modified version (K-L-PTD1) were able to eliminate GFP labeled intracellular *S. aureus* strain UAMS-1 from murine calvaria (Fig. 3). Surviving *S. aureus* UAMS-1 were detected by confocal microscopy of sectioned calvaria labeled with DAPI (Fig. 3A) and quantified (Fig. 3B). Both triple fusion K-L and K-L-PTD1 reduced the number of live bacteria recovered from sectioned calvaria (Fig. 3C), and in a femur wound model^30^ (Fig. 3D). These two animal models correlate well with the results of cultured osteoblasts in Fig. 2.

### PTD fusion to PGHs enhance biofilm eradication

**Figure 4:**
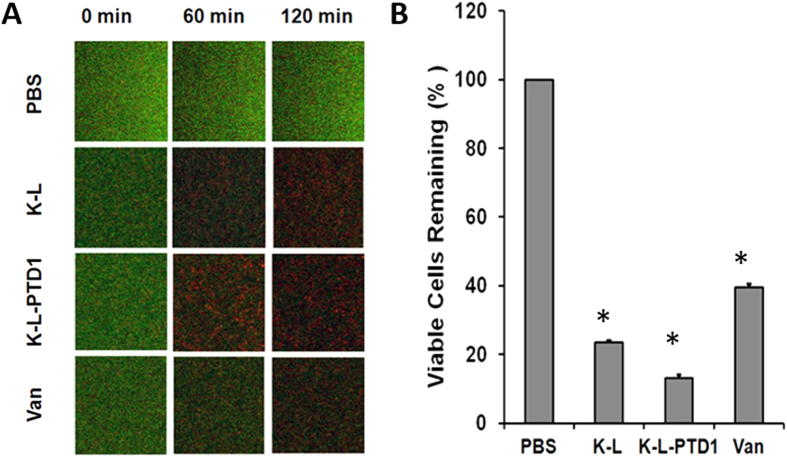
PTD1 enhances triple fusion K-L eradication of dynamic MRSA biofilms. (**A**) Confocal microscopy of biofilm with Live/Dead staining. Single 1 μm z-stack images in the middle of NRS382 (USA100) biofilms treated with 100 μg/ml (1.4 μM) triple fusion K-L, K-L-PTD1, (69 μM) vancomycin (Van) at a flow rate of 0.5 ml/min for 0, 60, or 120 minutes. Biofilms were pre-stained with the Live/Dead stain (see methods) and viewed with 20X magnification. (**B**) Bacterial viability. Analysis of bacterial viability in NRS382 dynamic biofilms based on mean fluorescent intensities of the Live/Dead viability stain when exposed to 100 μg/ml of PGH K-L (1.4 μM), K-L-PTD1 (1.4 μM), or vancomycin (69 μM) at a flow rate of 0.5 ml/min for 2 h and compared to PBS. Error bars represent SEM (N = 3) of three independent 100 × 100 pixel squares identically located in each biofilm z-stack. All values were found to be significantly different from PBS and each other using a two-tailed, unpaired, t-test; K-L vs. KL-PTD1 (p = 0.00064), K-L vs. vancomycin (p = 0.00036), and K-L-PTD1 vs. vancomycin (p = 0.000023), as indicated with an asterisk.

To determine whether modification with a PTD can impact biofilm clearance, triple-acting fusion K-L, K-L-PTD1, and vancomycin were tested for antimicrobial activity in a dynamic biofilm model with MRSA strain NRS382 (Fig. 4). At equal gram concentrations (100 μg/ml), all three had a pronounced effect on dynamic biofilms at 60 and 120 minutes post-treatment as visualized by Live/Dead™ viability staining (Fig. 4A). Despite a much reduced molar concentration compared to vancomycin [K-L (1.4 μM), K-L-PTD1 (1.4 μM), vancomycin (69 μM)] the PGHs were much more effective than vancomycin. Compared to controls (100% viable cells), vancomycin, triple-acting fusion K-L, and K-L-PTD1 reduced the viability in dynamic biofilms to 40%, 24%, and 13%, respectively, with K-L-PTD1 being significantly more effective than K-L (Fig. 4B). This quantification was based on the 1 μm confocal microscopy sections depicted in Fig. 4A. Compiling mean fluorescent intensities from the entire biofilm z-stack (i.e., 40 × 1 μm slices) yielded similar results (not shown).

### PGH-PTD fusion reduces *S. aureus* in mastitis model

**Figure 5:**
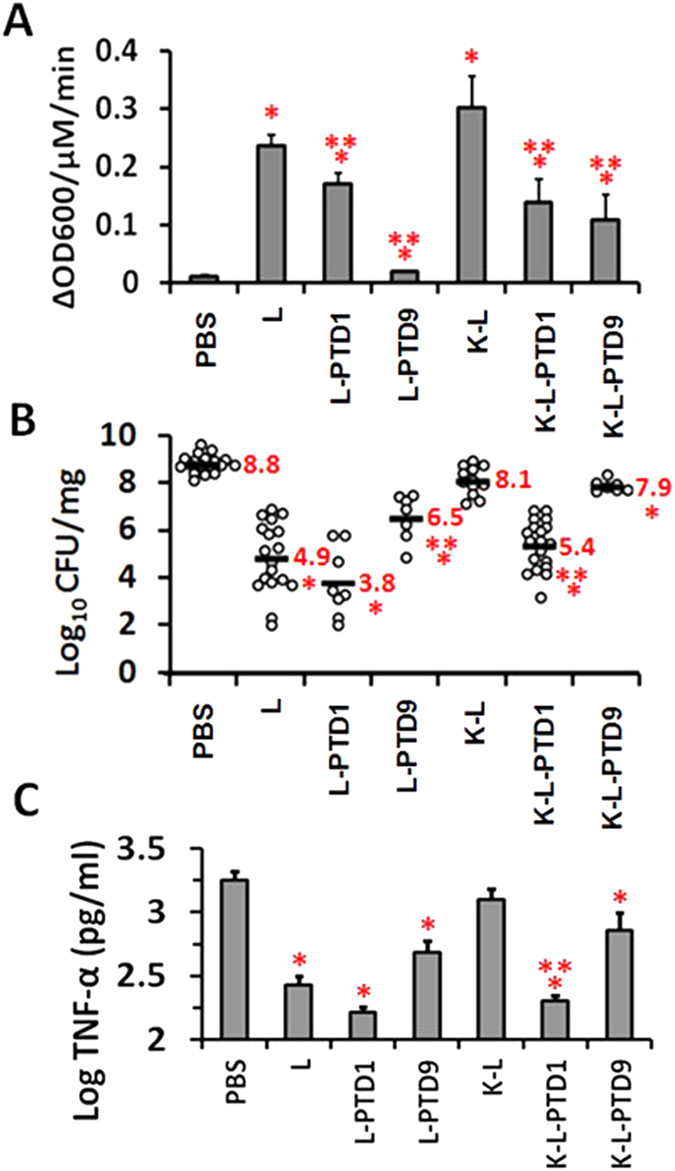
Triple-acting fusions can reduce *S. aureus* induced mastitis in a mouse model. (**A**) Specific activity of PGH fusion constructs *in vitro*. Turbidity reduction assay with *S. aureus* Newbould 305 resuspended in lysis buffer to an OD_600nm_ of 1.0 and treated with 1 μM final concentrations of each PGH. The maximum specific activity for three experiments is represented as an average with SEM error bars. Single asterisks indicate significant difference from buffer control (p < 0.05); double asterisks indicate significant difference from parental enzyme (p < 0.05). (**B**) Bacterial burden in mice with mastitis treated with recombinant PGHs. CFUs from infected murine mammary glands following a single treatment with 50 μl of 25 μM PGH (1.25 nmol). Each data point represents the average of duplicate bacterial platings. The horizontal bars represent the average CFU/mg for each group. (**C**) TNFα response in mice with mastitis that were treated with PGHs. Data represent a minimum of 7 measurements in duplicate. Log TNFα was used for statistical analysis. Single asterisk indicates a significant difference from buffer control (p < 0.05); double asterisk indicates significant difference from parental enzyme (p < 0.05).

To determine if addition of a PTD impacts the ability of a PGH to reduce the bacterial load in a murine model of mastitis, four constructs were tested. These were comprised of two different PTD domains fused to two different PGH constructs (L-PTD1, L-PTD9 and K-L-PTD1, K-L-PTD9) and were chosen based on factors including purification yield, solubility, stability (not shown), and our results in MAC-T cells (Fig. 2). In turbidity reduction assays triple-acting fusion K-L was slightly more effective against *S. aureus* strain Newbould 305 than lysostaphin, and the addition of PTD1 or PTD9 to either lysostaphin or triple-acting fusion K-L reduced the *in vitro* activity (Fig. 5A). Mice were challenged with 100 CFU *S. aureus* Newbould 305 in 50 μl buffer followed by 50 μl of the PGH (1.25 nmoles in PBS) or buffer (phosphate buffered saline; PBS) 30 min later. The dams were euthanized 18 h post infusion, mammary glands were aseptically dissected, and portions of the gland were used for bacterial load and TNFα determinations^21^. *In vivo,* lysostaphin was effective in reducing the bacterial load approximately 4 logs compared to controls (Fig. 5B), and reduced the TNFα concentration in the mammary tissue >6 fold after challenge (Fig. 5C). Fusion of PTD1 to lysostaphin (L-PTD1) further decreased the mean bacterial load and TNFα concentration, but these results were not significantly different from lysostaphin lacking the PTD. Although the L-PTD9 fusion was virtually inactive in the turbidity reduction assay (Fig. 5A), it was still capable of reducing bacterial load within the mouse mammary glands relative to the PBS buffer control, although it was significantly less effective than either L-PTD1 or lysostaphin alone (Fig. 5B,C). In contrast to our *ex vivo* data, triple-acting fusion K-L was not able to clear the mammary gland bacterial infection more than buffer alone. However, *S. aureus* clearance was significantly enhanced when triple-acting fusion K-L was fused to PTD1 (K-L-PTD1) yielding clearance of the mammary gland to a level equivalent to that of lysostaphin alone or L-PTD1 (Fig. 5B,C).

## Discussion

In response to the critical need for novel antimicrobials with reduced resistance development for treating MDR *S. aureus* we have engineered triple-acting staphylolytic PGHs. These constructs simultaneously degrade *S. aureus* cell walls, at three unique bonds in the highly repetitive PG structure. A primary advantage conferred by the PGH attack at the pathogen cell wall is the avoidance of most intracellular resistance mechanisms (e.g. efflux pumps). PGHs derived from bacteriophage endolysins have the added advantage of co-evolution with bacteria, allowing them to target bonds that the host cell cannot readily modify, yielding enzymes that are, in theory, inherently refractory to resistance development (resistance data for endolysins is reviewed in^31^). With multiple PG-degrading domains (glycosidase, endopeptidase, and amidase) in one molecule, there is considerable diversity and often near species-specificity of these activities, ensuring low selective pressure on unrelated, co-resident commensal strains, further reducing the potential for resistance development in non-targeted species. The PGHs have additional favorable qualities. These qualities include non-toxic^32^, biodegradable, effective on both biofilms^33^ and MDR strains^24^, are synergistic with antibiotics^34^, and thus hold great potential for treating MDR strains.

LysK was not as refractory to resistance development (measured by its MIC) as previously reported for *Streptococcus* and *Bacillus* endolysins^31^ and a staphylolytic fusion construct^35^ (in serial dilution plating assays). However, our strategy to reduce resistant strain development by creating triple-acting staphylolytic fusions was successful with very little resistance development for triple-acting fusion L-K *in vitro* (when tested against the lab strain *S. aureus* strain Newman), reflecting at least an order of magnitude improvement over either parental enzyme (alone or in combination). Triple-acting fusion L-K also reduced the bacterial load 5–10 fold better than either parental enzyme in a rat nasal decolonization model, with no *in vivo* resistance development detected. The observed 98% reduction in nasal bacterial load was virtually identical to the 98% CFU reduction achieved by mupirocin. Despite these successes, as proteins the PGHs must overcome a unique set of therapeutic hurdles.

A separate therapeutic hurdle is created in systemic infections where *S. aureus* can evade the host immune system (and most antibiotics) through intracellular localization and sequestration. Intracellular invasion has been reported for bovine mastitis [where *S. aureus* has been identified within mammary alveolar cells and macrophages isolated from milk^6^,^7^] and is implicated by the high frequency of MDR *S. aureus* in recurring osteomyelitis in “cured” blast wound victims from Middle Eastern war zones^10^. Toxic levels of conventional antibiotics are often required to treat classic intracellular pathogens^36^. To address this concern, we engineered our PGH constructs with 11 different PTDs and identified the optimal PTD domain(s) to facilitate import into multiple cultured cells. Our Initial data with lysostaphin indicated that a PTD was essential for eradication of intracellular *S. aureus* within both MAC-T cells and hBMECs. In contrast, triple-acting fusion K-L did not require a PTD to invade cultured bovine mammary cells or murine osteoblasts, and showed a similar efficacy with or without a PTD in bone infection models. Both LysK and triple fusion L-K were ineffective at intracellular eradication when fused to any of the eleven PTDs. Despite equivalent intracellular efficacy with either triple fusion K-L or construct K-L-PTD1 in cultured mammary cells, the latter showed almost 3 orders of magnitude greater CFU reduction *in vivo* in the mastitis model. The inconsistencies in these results between *ex vivo* and *in vivo* assays underline the complexity of cellular uptake mechanisms including the poorly defined role of PTD sequences, cargo protein sequences, and the variety of cellular uptake mechanisms that can be employed to achieve intracellular localization. Triple-acting fusions K-L (which showed highest efficacy in intracellular *S. aureus* eradication) and L-K (which showed highest efficacy in *S. aureus* nasal decolonization) both harbor virtually identical sequences, with only the order of the domains being rearranged, suggesting that an alternate tertiary structure likely contributes to their differential efficacy in these assays.

PGHs ability to reduce or eradicate static biofilms^33^ is an important advantage over conventional antibiotics, since biofilms are proposed to play a critical role in infectious disease^37^. There was an apparent benefit conferred by adding a PTD to the engineered triple fusion K-L in the eradication *S. aureus* dynamic biofilms. Again, the exact role of the PTD is unknown in this capacity.

Another therapeutic hurdle is the fact that as protein, PGHs are potentially antigenic and might engender host immune responses in a manner similar to that seen in phage-based therapies^38^. However, PGHs show minimal immunogenicity in mammals, and adverse responses have not been reported. Bovine intramammary infusions of lysostaphin resulted in detectable levels of specific antibodies only after 18–21 treatments. The antibodies were not neutralizing, nor did they elicit observable effects on the host animal or eliminate the antimicrobial properties of lysostaphin^39^. Serum antibodies raised to phage endolysins specific to *Bacillus anthracis, Streptococcus pyogenes,* or *Streptococcus pneumoniae* slowed, but did not inhibit microbial killing *in vitro*^31^,^40^. There is also a concern for proinflammatory components released from lysed bacteria^41^. However, adverse immune responses have not been observed in mouse models for an array of systemically delivered staphylolytic enzyme constructs. In fact, seven out of nine endolysins provided 100% protection from MRSA bacteremia versus 20% survival at 48 hours post infection in buffer or oxacillin treated animals^42^. Furthermore, a reduced TNFα response (to near baseline) when mouse mammary glands challenged with *S. aureus* were treated with our triple-acting fusion PGHs indicates a reduced inflammatory response compared to untreated controls.

The hurdles to commercialization of PGH antimicrobials are non-trivial. Still to be addressed are the physicochemical and pharmacokinetic aspects of PGH treatments [which are likely to be significant, considering the potential for intracellular transport]. Production hurdles are also expected when producing protein therapeutics. Despite these hurdles, the ability to engineer a PGH antimicrobial with qualities not readily achievable with conventional antibiotics (refractory to resistance development, biofilm eradication, treatment of intracellular and MDR pathogens) begs the question: What other desirable traits could be engineered into PGH antimicrobials? In the absence of an approved PGH therapeutic, we hope that a continued demonstration of the versatility of engineered PGHs will lower the threshold to commercialization for this new and much needed class of antibacterials.

## Materials and Methods

All bacterial strains, culture conditions, details of *E. coli* expression constructs, purification, and PGH activity characterization (turbidity reduction, plate lysis, zymogram and MIC assays) are described previously^25^ (Supplementary Information Materials and Methods). Resistance development assays (liquid- and plate lysis-based) are essentially as described previously^43^ (Supplementary Information Materials and Methods). Static and dynamic biofilm reduction assays are as previously described^44^ (SI Materials and Methods). Intracellular *S. aureus* eradication assays from three different labs are based on previously reported protocols^45^ (Supplementary Information Materials and Methods). Confocal microscopy to demonstrate intracellular colocalization of both fusion enzymes and *S. aureus* within cultured MAC-T cells is described in Supplementary Information Materials and Methods. Cytotoxicity studies were performed on MAC-T cells per the *in situ* method of Perry *et al.*^29^. Four previously described animal models (*in vivo* rat nasal colonization^46^, *ex vivo* murine cavalaria, *in vivo* femur osteomyelitis^30^ and *in vivo* murine mastitis^47^ were used to demonstrate efficacy of the fusion constructs (Supplementary Information Materials and Methods). All animal experiments were conducted in accordance with protocols approved by the appropriate Institutional Animal Care and Use Committees.

## Additional Information

**How to cite this article**: Becker, S. C. *et al.* Triple-acting Lytic Enzyme Treatment of Drug-Resistant and Intracellular *Staphylococcus aureus. Sci. Rep.* **6**, 25063; doi: 10.1038/srep25063 (2016).

## Supplementary Material

Supplementary Information

## Figures and Tables

**Table 1:** Protein transduction domains.

PTD	Sequence	Ref.
1	RQIKIWFQNRRMKWKK	48
2	WEAKLAKALAKALAKHLAKALAKALKACEA	49
3	MVTVLFRRLRIRRASGPPRVRV	50
4	KLALKLALKALKAALKLA	51
6	RRQRRTSKLMKR	52
7	LLIILRRRIRKQAHAHSK	53
8	RRRRRRRR	54
9	GRKKRRQRRRPPQ	55
10	GWTLNSAGYLLGKINLKALAALAKKIL	56
11	AGYLLGKINLKALAALAKKIL	57
12	RKKRRQRRR	55
