# Supplementary material for: Triple-acting Lytic Enzyme Treatment of Drug-Resistant and Intracellular Staphylococcus aureus
Source: Sci Rep. 2016 Apr 28;6:25063. doi: 10.1038/srep25063 (PMC4848530; doi:10.1038/srep25063)

# Triple-acting Lytic Enzyme Treatment of Drug-Resistant and Intracellular *Staphylococcus aureus*.

Stephen C. Becker[a,*] Dwayne R. Roach[a,k,*], Vinita S. Chauhan[b], Yang Shen[c,i], Juli Foster-Frey[a], Anne M. Powell[a], Gary Bauchan[a], Richard A. Lease[a,h], Homan Mohammadi[a], William J. Harty[a], Chad Simmons[a], Mathias Schmelcher[a,i], Mary Camp[a], Shengli Dong[e,j], John R. Baker[e], Tamsin R. Sheen[f], Kelly S. Doran[f], David G. Pritchard[e], Raul A. Almeida[g], Daniel C. Nelson[c], Ian Marriott[b], Jean C. Lee[d], and David M. Donovan[a§]

**Affiliations and addresses:**

[a] ARS, USDA, 10300 Baltimore Ave, Beltsville, MD, [b] Biology, Univ. North Carolina, Charlotte, Charlotte, NC, [c] Institute for Bioscience and Biotechnology Research, University of MD, Rockville, MD and Department of Veterinary Medicine, University of MD, College Park, MD, [d] Channing Laboratory, Department of Medicine, Brigham and Women's Hospital and Harvard Medical School, Boston, MA, [e]Biochemistry, Univ. Alabama, Birmingham, Birmingham, AL, [f] Biology, San Diego State University, San Diego, CA, [g]University of Tennessee, Knoxville, TN

Current Address: [h] Dept. of Chemical & Biomolecular Engineering, The Ohio State University, Columbus, OH, [i] Institute of Food, Nutrition and Health, ETH Zurich, Zürich, Switzerland, [j] Department of Biochemistry and Molecular Biology, Louisiana State University Health Sciences Center, New Orleans, LA, [k]Unité de Biologie Moléculaire du Gène chez les Extrêmophiles Département de Microbiologie, Institut Pasteur, Paris, France

*Authors SCB and DRR share first authorship.

**SI Materials and Methods:**

**Bacterial Strains.** Mastitis isolates *S. aureus* Newbould 305, *Staphylococcus hyicus* MP01, *Staphylococcus chromogenes* MP02, *Staphylococcus simulans* MP03, *Staphylococcus epidermidis* MP04, *Staphylococcus xylosus* MP05, and *Staphylococcus warneri* MP06 were gifts from Max Paape (USDA, Beltsville). Strain *S. aureus* SA113 was provided by Andreas Peschel (University of Tübingen, Germany). *S. aureus* strains Newman [17], ALR [17], UAMS-1 [12], and ISP479C [22] were described previously. The remaining strains (designated with NRS numbers) were obtained from Network on Antimicrobial Resistance in *S. aureus* (NARSA) repository. The antibiotic resistance profiles of the strains are listed in **Supplementary Table 1**.

**PGH Expression Constructs:** A plasmid harboring the gene encoding the 247 amino acid mature (secreted) lysostaphin (construct L) (UniProtKB/Swiss-Prot #: P10547.2) was a gift from David Kerr, University of Vermont. The staphylococcal phage K endolysin cDNA encoding the 495 amino-acid *lysK* gene product (construct K) (Genbank AAO47477.2) was provided by Paul Ross [15]. All constructs were performed in *E. coli* DH5α (Invitrogen, Carlsbad, CA) via PCR cloning [3] or conventional DNA fragment isolation and ligation using standard methods, and were verified by DNA sequencing. All constructs harbor a C-terminal $His_6$ tag derived from pET21a (EMD Biosciences, San Diego, CA). All PCR primers and plasmids are listed in **Supplementary Table 2**.

Triple fusion K-L encoded by expression vector pSB1101 was created from the *lysK* truncation LysK390' expression vector pSB0301, previously described [2]. The

lysostaphin fragment fused to the 3’ end of pSB1101 was amplified with primers LysoSalIF and LysoXhoIR, digested with XhoI and SalI, and introduced into pSB0301 linearized at the XhoI site, generating pSB1101.

Triple fusion L-K encoded by expression vector pSB1801 was created in several steps. The lysostaphin gene from plasmid p5301 was truncated by PCR-amplifying the M23 peptidase domain with the primers LysoAD155XhoIR and LysoAA1NdeI F and introducing this PCR product into NdeI + XhoI-digested pET21a, thereby generating pSB1701. A second intermediate construct was produced by amplification of the lysostaphin SH3b domain from plasmid template p5301 with the primers LysoSH3b SalIF and pET21a StyIR, digesting with SalI + StyI, and introducing the amplified fragment into XhoI+ StyI-digested pSB0301, thereby generating plasmid pSB1001. The final triple fusion L-K construct was generated by introducing the PCR product generated by amplification of the template pSB1001 with the primers LysKaaSalF and pET21aStyIR (harboring the two lytic domains of LysK) into XhoI + StyI-digested pSB1701, thereby generating pSB1801.

Fusion of PGH to PTD sequences was accomplished by reverse translation of the 12 PTD sequences (**Table 1**) into nucleotide sequences (with an *E. coli* codon usage bias), followed by commercial synthesis (Genscript, Piscataway, NJ). The individual sequences were inserted at the XhoI site of pET21a such that each PTD coding sequence was in frame with the $His_6$ coding sequences of the vector. The four PGH sequences (encoding mature lysostaphin, *lysK*, and triple fusions K-L and L-K) were introduced into these PTD-$His_6$ encoding vectors via standard procedures following restriction enzyme digests of the parental PGH vectors (described above) at unique

sites (XbaI or NdeI and XhoI) to generate DNA fragments harboring the entire PGH coding region, and ligation of these fragments into similarly digested pET21a vectors harboring the 12 individual PTD sequences.

**Protein expression and purification.** Recombinant PGHs were expressed in *E. coli* BL21 (DE3) (Novagen) grown to mid-log phase at 37°C in modified LB broth (15g tryptone, 8g yeast extract, 5g NaCl per liter, pH 7.8) with shaking, induced with 1mM IPTG, and expressed for 20 h at 10ºC. Cells were disrupted by sonication and purified via nickel affinity chromatography (Ni-NTA-agarose), per the manufacturer's instructions (Qiagen, Carlsbad CA) to >95% purity as described previously [3]. For PGHs used in animal and cell culture experiments, endotoxin was removed via Triton X-114 washes of protein on Ni-NTA columns [18]. Representative random samples were evaluated for endotoxin content via the *Limulus* amoebocyte lysate assay (LAL QCL-1000, Lonza, Walkersville, MD) and shown to be <5 Units/ml (e.g. for nasal colonization <5 Units/~40 mg protein) endotoxin after purification.

**Preliminary characterization of PGH activity.** SDS-PAGE, zymogram, turbidity reduction and plate lysis assays were performed as described previously [2] with minor modifications as follows. For SDS-PAGE and zymogram analysis, 1 µg of the purified PGH proteins and Kaleidoscope protein standards (Invitrogen, Carlsbad, CA) were analyzed by 15% SDS-PAGE. SDS-PAGE and zymogram gels were prepared and electrophoresed in parallel. Zymograms incorporated embedded cells equivalent to 300 ml of mid-logarithmic phase ($OD_{600nm}$ 0.4-0.6) *S. aureus* Newman into the SDS-PAGE matrix. The SDS-PAGE gels were stained with Coomassie blue using standard protocols, and zymograms were washed twice in excess water for 30 min to remove

SDS and incubated for <1 h at room temperature in water until cleared zones developed.

For the plate lysis assays, purified enzymes were serially diluted in saline lysis buffer (SLB; 150 mM NaCl 10 mM Tris buffer, pH 7.5) with 15% glycerol, to yield concentrations of 100, 10, 1, and 0.1 pmoles/10 µl. *S. aureus* Newman was cultivated in tryptic soy broth (TSB) to an $OD_{600\ nm}$ = 0.4 – 0.6. The bacterial cells were harvested and diluted to yield a suspension with an $OD_{600\ nm}$ of 0.1. Tryptic soy agar (TSA) plates were flooded with 3 ml of the bacterial cell suspension. Excess culture was removed, and the plates air dried at room temperature for ~30 min in a laminar flow hood. 10 µl of each PGH dilution was then spotted onto the air-dried lawn, allowed to air dry, and the culture plates incubated overnight at 37°C. The following day, plates were evaluated visually and photographed.

**Resistance development assays.** *MIC repeated exposure method.* MIC determinations were performed with *S. aureus* strain Newman. 100 µl of *S. aureus* strain Newman culture that survived at ½ the MIC (the first well with visible growth) for each round was inoculated into 5 ml TSB and cultivated to mid-log phase growth. This culture was used as the inoculum for the next round of MIC exposure (overnight growth), and the cycle was repeated for 10 rounds. Cells recovered from the well that represented the ½ MIC concentration on round 10 were confirmed to be *S. aureus* by PCR[13]. *Plate lysis method.* Bacterial cells were scraped from a sub-lethal (not fully cleared) spot from a plate lysis assay (described above), and these 'exposed' cells were used to inoculate 5 ml of TSB and grown for 4-6 hours to generate a new culture and lawn for subsequent exposure. Bacteria were passaged in this assay for up to 10

consecutive days, at which time the *S. aureus* were tested in MIC assays. Plate lysis repeated exposure experiments were performed in duplicate.

**Determination of PGH minimum inhibitory concentrations (MIC).** The MIC of each protein for multiple *S. aureus* strains was determined as previously described [10] with the following modifications. Enzymes were serially diluted two fold across a 96 well plate such that after dilution 50 µl of the enzyme solution in 300 mM NaCl, 50 mM $NaH_2PO_4$, 30% glycerol pH 7.5, remained in each well. To each well was added 50 µl of TSB and 100 µl of *S. aureus* Newman in TSB (diluted to 5 x $10^6$ cells/ml). The plates were incubated 20 h at 37ºC and read with a 96 well plate reader. MIC determination values were reported as the median of $\geq$4 replicates (**Supplementary Table 1**).

**Confirmation of three lytic PGH enzyme activities in triple fusion constructs**. LysK is a 495 amino acid protein with a C-terminal SH3b CBD (SH3-5, Pfam[8] PF08460) and two lytic domains (**Fig. 1A**). The N-terminal lytic domain is a cysteine, histidine-dependent amidohydrolase/peptidase (CHAP) domain[19] (Pfam PF05257), and the second (internal) lytic domain has been classified as an amidase-2 domain (PFAM PF01510). Both lytic domains are active and show specific cleavage sites on purified *S. aureus* PG [2,7]. The CHAP endopeptidase activity cleaves between the D-alanine of the stem peptide and the first glycine of the pentaglycine cross-bridge peptide, and the amidase-2 domain harbors an N-acetylmuramoyl-L-alanine amidase activity that cleaves between the N-acetylmuramic acid of the polysaccharide strand and L-alanine of the stem peptide (**Supplementary Fig. 2**). Mature lysostaphin is a 246 amino acid

protein with a single enzymatic domain (**Fig. 1A**), an N-terminal M23 glycyl-glycine endopeptidase (Pfam PF01551) that cleaves between the second and third, or third and fourth, glycines of the *S. aureus* pentaglycine cross bridge (**Supplementary Fig. 2**) [5]. Like many staphylococcal PGH and phage endolysins[4], the bacteriocin lysostaphin also harbors a C-terminal SH3b CBD (SH3-5, Pfam PF08460).

Purified *S. aureus* PG was digested by triple fusions created with LysK and lysostaphin, and the products were examined via Electrospray Ionization Mass Spectrometry (ESI-MS). For all triple fusion constructs examined, the PG digestion products yielded peaks at m/z 702, 645, 588, 531, and 474 that are identical to the peaks obtained with a double digestion with both enzymes (including triple fusions described previously [7]). Representative peaks are shown in **Supplementary Figure 2** for triple fusion K-L. Peaks observed at m/z = 588 ($A_2QKG_3$) and 531 ($A_2QKG_2$) require cleavage by all three domains. A previously reported head to tail of full length LysK and lysostaphin (with reduced activity compared to triple fusion K-L and L-K) was also reported to maintain all three PG cut sites [7].

**Static biofilm reduction**. Biofilms were produced as previously described [9] with the following modifications. *S. aureus* strains were grown overnight in 5 ml TSB supplemented with 0.25% D(+)-glucose without shaking at 37°C. The culture was diluted 1/200 in fresh culture medium, and 200 µl was added to each well of a 96 well microtiter plate and incubated without shaking for 24 h at 37°C. Biofilms in the wells were washed twice with 200 µl SLB, treated with 50 µl PGH in SLB at concentrations indicated (**Supplementary Fig. 3**) for 1 h at room temperature, and washed twice with 100 µl SLB. Adherent bacteria were fixed with 200 µl 95% ethanol-5% glacial acetic

acid for 20 min, washed once with water, and stained with 100 µl 0.4% crystal violet for 15 min at room temperature. Excess stain was removed with 3 water washes. Bound stain was resolubilized in 33% acetic acid, and 20 µl was transferred to a 96 well plate containing 180-µl water. The $OD_{590nm}$ was determined in a Spectra Max plate reader. Percent reduction represents the difference in absorbance between biofilms exposed to buffer only and experimental wells that were exposed to the PGHs.

**Dynamic biofilm reduction.** To develop dynamic *S. aureus* NRS382 biofilms, a 1:10 dilution of an overnight culture (TSB) was inoculated into a single-chamber or three-channel Stovall flow cell (IBI Scientific) with fresh TSB at 37ºC. The inoculum was static for the first hour to allow initial bacterial attachment to the glass cover slip. The flow of fresh TSB was then started at a rate of 0.5 ml/min for up to 48 h to allow for development of mature biofilms.

In order to visualize the viability of *S. aureus* NRS382 biofilms, each chamber of the flow cell was fluorescently labeled by the Live/Dead BacLight™ staining kit (Invitrogen, Carlsbad, CA) for 1 h. The flow cells were then treated with either buffer (PBS), 100 µg/ml of vancomycin, triple fusion K-L or K-L-PTD1, all in PBS with a flow rate of 0.5 ml/min. At 0, 60, and 120 minutes, z-stacks of horizontal-plane images of biofilms were obtained with a 20X/1.3 objective lens by a Zeiss 710 confocal laser scanning microscope. The total z-stack image series contained 40 x 1 µm sections. To quantify the viability of *S. aureus* NRS382 biofilms at various time points in the presence of different treatments, one image at the center of z-series (i.e. center of the biofilm) for each time point was selected to represent each treatment group. Three squares representing 100 x 100 pixels were drawn in each image, and the mean intensity of live

(green channel) and dead (red channel) cell populations was calculated by Zen 2010 digital imaging software (Carl Zeiss). Percentage of live cells in the dynamic biofilm was calculated using a calibration “standard” curve that assigned a fluorescence intensity to live *S. aureus* NRS382 cells, over a range of cell concentrations. This allowed a direct translation of the mean fluorescent intensities to a percentage of live cells. The percentage of live cells remaining in biofilms after various treatments was statistically analyzed by an unpaired t-test.

**Intracellular *S. aureus* eradication assay.** Three similar intracellular *S. aureus* eradication from three different labs were optimized for (A) a cultured bovine mammary epithelial cell line (MAC-T, Nexia Biotechnologies, Quebec, Canada) [1], (B) primary C57BL/6 mouse-derived osteoblasts (mOBs)[14] or (C) human brain microvascular endothelial cells (hBMEC)[23]. MAC-T cells were grown in 24-well dishes, infected with 1.24 x $10^7$ to 6.47 x $10^7$ *S. aureus* Newbould 305 (a known bovine mastitis causative agent) at an MOI of 28 to 209 for 2 h at 37°C in Dulbecco’s Modified Eagle’s Medium (DMEM) with no antibiotics. Osteoblasts were grown in 6 well plates as described earlier [6] and infected with the *S. aureus* strain UAMS-1 at an MOI of 100:1 for 2 h at 37°C in osteoblast growth medium in the absence of antibiotics. hBMEC were cultured in the absence of antibiotics and infected with ~1 x $10^6$ (MOI = 10) *S. aureus* strain ISP479C[16], in RPMI culture media with 10% fetal bovine serum (FBS) for 2h at 37ºC, as described previously [22]. Following 3X PBS washes to remove extracellular *S. aureus*, the infected eukaryotic cell cultures (A-C, above) were exposed to gentamicin (A) 100 µg/ml, 1h (B) 50 µg/ml, 15 min, or (C) 100 µg/ml, 30 min to kill extracellular *S. aureus*. Subsequently, cells (A) and (C) were washed 3X with PBS to remove gentamicin,

whereas cells (B) were cultured in the presence of 25 µg/ml gentamicin for 24 h. Purified PGH proteins were added to (A) MAC-T cells at 25 µg in 1 ml DMEM for 2.5 h, (B) mOBs at 5 µg/ml for 2 h, or (C) the hBMECs at 5 µg/ml for 1 h. Gentamicin-only controls received (A) 100 µg/ml, (B) 25 µg/ml, or (C) 100 µg/ml gentamicin in place of the PGH construct. The cultures were finally washed 3X with PBS and examined microscopically (to determine if there had been cell lysis) prior to being trypsinized with 0.05% trypsin + 0.025% Triton X-100 in (A and C) PBS or (B) water. The cell lysate was subjected to serial dilution plating, and the resultant CFUs were counted and normalized to the gentamicin control for each assay. CFU numbers in the gentamicin only treated group were in the range of (A) $3.26 \times 10^4$ to $6.19 \times 10^5$, (B) 6-10 $X 10^3$ or (C) 2-4 $\times 10^5$ per well. All analyses were performed with one factor ANOVA ($\alpha$=0.05) and t-Test post hoc analysis with Šidák correction to $\alpha$, ($\alpha$=0.05) for multiple comparisons, comparing each PGH treatment to the gentamicin only treatment.

**Confocal Microscopy of MAC-T cells.** A 10 ml TSB overnight culture of *S. aureus* strain Newbould was washed, resuspended in 10 ml DMEM without FBS, vortexed rigorously and pelleted prior to resuspension in fresh 10 ml DMEM without FBS to an $OD_{600}$= 0.2. 100 µl of 1 mg/ml AlexaFluor-488 labeled wheat germ agglutinin (WGA; Life Technologies) was added to 10 ml *S. aureus* 305 in DMEM. The cells were incubated at 37ºC for 30 min, washed 6X and resuspended in DMEM without FBS.

Confocal fluorescent microscopy was performed for *in situ* detection of internalized *S. aureus* and PGH particles in bovine MAC-T cells cultivated on uncoated 50 mm glass bottom dishes (MatTek Corporation, Ashland, Massachusetts) in high-glucose DMEM

(HyClone, Logan, Utah) supplemented with 10% (v/v) heat-inactivated FBS (Atlanta Biologicals, Lawrenceville, Georgia) at 37°C in a humidified 5% $CO_2$ incubator. After 18 h of culture, when MAC-T cells were about 90-95% confluent, the existing medium was removed and replaced with 1 ml DMEM without FBS containing AlexaFluor488-labeled *S. aureus* cells. Samples were incubated at 37ºC for 30 min, washed 6X with DMEM without FBS, and 1 ml media was added to each well of a 24-well plate. After 2 h, MAC-T cells were washed 3X in DMEM without FBS, exposed to 200 µg/ml gentamicin in DMEM for 30 min and washed an additional 3X to remove extracellular *S. aureus*. MAC-T cells were exposed to 50 µg/ml of construct K-L-PTD 1 labeled with AlexaFluor 610-X per the manufacturer's protocol (Life Technologies Corporation) for 2 h before 3X DMEM washes. DMEM was replaced with TL HEPES (bio.Lonza.com; solution # 04-616) and a drop of NucBlue Fixed Cell Stain (Life Technologies Corporation) was applied to the MAC-T cells prior to imaging.

**Animal Models:** All animal experiments were conducted in accordance with protocols approved by the appropriate Institutional Animal Care and Use Committees.

**Rat model of nasal colonization.** The rat nasal colonization model is an adaptation of the mouse nasal colonization model that was described previously [20]. Wistar rats (5 to 6 wks of age) were obtained from Charles River Laboratories (Wilmington, MA) and given food and water *ad libitum*. The animals were housed two per cage in a modified barrier facility under viral antibody-free conditions. The rats were given drinking water containing streptomycin sulfate (0.5 g/liter; Sigma) 1 day prior to bacterial inoculation and for the course of the experiment. The drinking water and cages were changed three times per week. Rats were restrained briefly for inoculation in a DecapiCone bag

(Braintree Scientific, Braintree, MA) and inoculated intranasally with 10 µl of $10^7$ CFU *S. aureus* strain ALR (Sm-resistant) on day 0. The rats were treated intranasally twice a day (6-7 h apart) on days 5, 6, and 7 with 200 µg of either lysostaphin (AMBI, Tarrytown, NY) or recombinant PGHs in 20 µl buffer. Control rats received buffer alone. On day 10 the rats were euthanized; the area around the nasal region was wiped with 70% isopropyl alcohol, and the nasal tissue was excised and homogenized on ice in 600 µl TSB. Quantitative cultures of the homogenates were prepared by plating duplicate 100 µl aliquots on TSA plates containing 0.5 mg/ml Sm (to recover the *S. aureus* strain used for inoculation) and one blood agar plate (to evaluate the total nasal flora). The number of *S. aureus* colonies from quantitative plate counts was used to estimate the CFU/nose for each rat. The results from a three independent experiments were combined. For rat nasal colonization experiments, significant ($P < 0.05$) differences between the median values of quantitative culture results for different rat groups were compared to the control group by the Mann-Whitney test (InStat; GraphPad Software).

***Ex-vivo* calvaria infections with *S. aureus*.** Calvaria were isolated from neonatal mice and infected with UAMS-1 strain of *S. aureus* ($1 \times 10^6$ CFU/calvaria) for 2 h at 37° C in osteoblast growth medium in the absence of antibiotics. Following 3X washes with PBS, calvaria were exposed to 50 µg/ml of gentamicin for 15 min to kill extracellular bacteria and further cultured in gentamicin containing osteoblast growth media. After 24 h, calvaria were treated with PGHs (5 µg/ml) or gentamicin only for 2 h. Lysis buffer containing 0.05% trypsin and 0.25% Triton X-100 was used to homogenize the calvaria, the homogenates were quantitatively plated on LB agar plates, and the CFU were

enumerated after 24 h. The data represent triplicate determinants of two separate experiments.

**Fluorescent microscopy for *ex-vivo* calvaria infections with *S. aureus*.** Calvaria were isolated and infected as described above using GFP-tagged *S. aureus* strain UAMS-1. Two hours post infection, calvaria were washed with PBS followed by a 15 min treatment with 50 µg/ml gentamicin to kill extracellular bacteria. 24 hours post infection, calvaria were treated with 5 µg/ml PHGs or gentamicin only control for 2 h and embedded in Tissue-Tek O.C.T compound (Sakura, Torrance, CA). Tissues were sectioned and nuclei were counter stained with DAPI (Life Technologies, Grand Island, NY). GFP and DAPI in uninfected and infected sections were visualized under a fluorescent microscope at 20X. To quantify the results, fluorescence intensity from the sections was measured using ImageJ software and defined as arbitrary fluorescence intensity units.

**Murine model of staphylococcal osteomyelitis.** We utilized a previously described [12] murine model of staphylococcal osteomyelitis that reproduces the clinical and gross pathological phases of inflammatory bone diseases, such as human post-traumatic osteomyelitis. C57BL/6J mice were anesthetized with isoflurane, and the femur was surgically exposed. A trough was drilled through the bone cortex by use of a high-speed drill with a round burr. Damaged bone sites were inoculated with *S. aureus* (1 x $10^3$ CFU) in agarose beads. Agarose beads containing *S. aureus* were prepared as follows: 1.4% low melt agarose (Invitrogen, Carlsbad, CA) was cooled to 40–42°C prior to the addition of bacteria. This mixture was added to mineral oil, vigorously stirred, and cooled rapidly on ice. The resulting agarose beads were washed and stored on ice

prior to bone application. This method of application induces local infection in bone tissue but markedly reduces the risk of systemic bacterial infection. The muscle fascia and surgical incision were closed, and the disease was allowed to proceed for 24 h. Mice were either untreated or treated intramuscularly at the site of infection with PGHs (triple fusion K-L or K-L-PTD1; 5 mg/kg) given twice in a 24 h period. Animals were euthanized, and the femurs were removed, homogenized in lysis buffer (0.05% trypsin and 0.25% Triton X-100 in water), and plated on LB agar plates. The data shown are the average normalized colony counts per ml of lysis buffer and represent the results of two separate experiments (n = 6). Asterisks represent statistical significance as determined by one-way ANOVA followed by Tukey's posthoc test.

**Murine mastitis model.** To evaluate the efficacy of purified, individually administered PGHs in a murine model of bovine mastitis, female C57BL6/SJL mice were challenged by the intramammary route with 100 CFU *S. aureus* Newbould 305 in 50 µl buffer. A single 1.25 nmoles intramammary infusion of each desired PGH (50 µl of a 25 µM solution) or 50 µl PBS was given 30 min post infection [with up to six glands treated per animal, each gland receiving a unique treatment] as described earlier [11,21]. Dams were euthanized 18 h post infusion, the mammary glands were aseptically dissected, and portions of the gland were used for determination of bacterial load and TNFα concentration, as described previously [21].

**Statistical analysis**. The variables were analyzed as two-factor mixed models with Treatment as the factor and Mouse as random Blocks. This model ignores within mouse correlations. Log(TNFα) was used. The variance grouping technique was used for log CFU and log TNFα to correct for variance heterogeneity. Means comparisons were

done with Sidak adjusted p-values so that the experiment-wise error was 0.05.

Log(TNFα) was back-transformed to the original units for graphing.

**SI References:**


1 R. A. Almeida*, et al.*, *Staphylococcus aureus* invasion of bovine mammary epithelial cells, J Dairy Sci **79**(6), 1021 (1996).

2 S. C. Becker*, et al.*, LysK CHAP endopeptidase domain is required for lysis of live staphylococcal cells, FEMS Microbiol. Lett. **294**(1), 52 (2009).

3 S. C. Becker, J. Foster-Frey, and D. M. Donovan, The phage K lytic enzyme LysK and lysostaphin act synergistically to kill MRSA, FEMS Microbiol Lett. **287**(2), 185 (2008).

4 S. C. Becker*, et al.*, Differentially conserved staphylococcal SH3b_5 cell wall binding domains confer increased staphylolytic and streptolytic activity to a streptococcal prophage endolysin domain, Gene. **443**(1-2), 32 (2009).

5 H. P. Browder*, et al.*, Lysostaphin enzymatic mode of action, Biochem Biophys Res Commun **19**(383), 389 (1965).

6 V. S. Chauhan and I. Marriott, Differential roles for NOD2 in osteoblast inflammatory immune responses to bacterial pathogens of bone tissue, J. Med. Microbiol. **59**(Pt 7), 755 (2010).

7 D. M Donovan*, et al.*, Peptidoglycan hydrolase enzyme fusions for treating multi-drug resistant pathogens., Biotech International **21**(2), 6 (2009).

8 R. D. Finn*, et al.*, The Pfam protein families database, Nucleic Acids Res. **36** (Database issue), D281-D288 (2008).

9 M. Gross*, et al.*, Key role of teichoic acid net charge in *Staphylococcus aureus* colonization of artificial surfaces, Infect. Immun. **69**(5), 3423 (2001).

10 R. N. Jones*, et al.*, Susceptibility Tests: Microdilution and Macrodilution Broth Procedures., in *Manual of clinical microbiology,* (eds. Balows, A. et al.) 972-977. (American Society for Microbiology, Washington D.C., 1985)

[11] D. E. Kerr, *et al.*, Lysostaphin expression in mammary glands confers protection against staphylococcal infection in transgenic mice, Nat. Biotechnol. **19**(1), 66 (2001).

[12] I. Marriott, *et al.*, Osteoblasts produce monocyte chemoattractant protein-1 in a murine model of *Staphylococcus aureus* osteomyelitis and infected human bone tissue, Bone. **37**(4), 504 (2005).

[13] F. Martineau, *et al.*, Species-specific and ubiquitous-DNA-based assays for rapid identification of *Staphylococcus aureus*, **36**(3), 618 (1998).

[14] S. H. McCall, *et al.*, Osteoblasts express NLRP3, a nucleotide-binding domain and leucine-rich repeat region containing receptor implicated in bacterially induced cell death, J Bone Miner. Res. **23**(1), 30 (2008).

[15] S. O'Flaherty, *et al.*, The recombinant phage lysin LysK has a broad spectrum of lytic activity against clinically relevant staphylococci, including methicillin-resistant *Staphylococcus aureus*, J. Bacteriol. **187**(20), 7161 (2005).

[16] P. A. Pattee, Distribution of Tn551 insertion sites responsible for auxotrophy on the *Staphylococcus aureus* chromosome, J. Bacteriol. **145**(1), 479 (1981).

[17] G. Regev-Yochay, *et al.*, Interference between *Streptococcus pneumoniae* and *Staphylococcus aureus*: In vitro hydrogen peroxide-mediated killing by *Streptococcus pneumoniae*, **188**(13), 4996 (2006).

[18] P. Reichelt, C. Schwarz, and M. Donzeau, Single step protocol to purify recombinant proteins with low endotoxin contents, Protein Expr. Purif. **46**(2), 483 (2006).

[19] D. J. Rigden, M. J. Jedrzejas, and M. Y. Galperin, Amidase domains from bacterial and phage autolysins define a family of gamma-D,L-glutamate-specific amidohydrolases, Trends Biochem. Sci. **28**(5), 230 (2003).

[20] A. C. Schaffer, *et al.*, Immunization with *Staphylococcus aureus* clumping factor B, a major determinant in nasal carriage, reduces nasal colonization in a murine model, Infect. Immun. **74**(4), 2145 (2006).

[21] M. Schmelcher, *et al.*, Chimeric phage lysins act synergistically with lysostaphin to kill mastitis-causing *Staphylococcus aureus* in murine mammary glands, Appl. Environ. Microbiol. **78**(7), 2297 (2012).

[22] T. R. Sheen, *et al.*, Penetration of the blood-brain barrier by *Staphylococcus aureus*: contribution of membrane-anchored lipoteichoic acid, J. Mol. Med. (Berl). **88**(6), 633 (2010).

[23] M. F. Stins, *et al.*, Binding characteristics of S fimbriated *Escherichia coli* to isolated brain microvascular endothelial cells, Am. J. Pathol. **145**(5), 1228 (1994).

**Supplementary Figure Legends:**

**Supplementary Figure 1. ESI-MS of purified *S. aureus* PG digested with triple fusion K-L.** Top: *S. aureus* PG schematic with enzyme cut sites of the parental enzymes indicated. Bottom: ESI-MS spectrum of products obtained after triple fusion K-L digestion of purified *S. aureus* PG. Major peaks observed are labeled with the possible compositions and schematic structures from the model above. Lyso, mature lysostaphin; GlcNAc, N-acetylglucosamine; MurNAc, N-acetyl muramic acid; D-iGln, isoglutamine (α-amidoglutamic acid).

**Supplementary Figure 2. PGH purity and *S. aureus* bactericidal assay. A. SDS-PAGE analysis** of 5 µg $His_6$-tagged PGHs purified by Ni-NTA chromatography. **B. Zymogram analysis** using whole *S. aureus* (Newman) cells embedded within the gel matrix. **C. Plate lysis analysis** of PGH constructs using live *S. aureus* (Newman) cell lawns clarified with indicated picomoles of each purified PGH. **D. Turbidity reduction analysis:** Determining the linear range of enzyme concentration. A twofold dilution series of each enzyme was used in a 30 min turbidity reduction assay on live *S. aureus* strain Newman cells to determine the enzyme concentrations that are within the linear range for each enzyme. Reaction conditions were 150 mM NaCl in SLB. Black, dark

grey, light grey, and white bars represent PGH constructs lysostaphin (L), LysK (K), triple fusion K-L, and triple fusion L-K, respectively, as named in **Fig. 1A**. Values are reported as the maximum rate $\Delta OD_{600nm}$/min ± SEM.

**Supplementary Figure 3. Effect of enzyme concentration on static biofilm clearance.** *S. aureus* SA113 biofilms were treated for 1 h with 100 µl of each enzyme individually or as equimolar mixtures of LysK and lysostaphin (white bars) in 300 mM NaCl, 50 mM $NaH_2PO_4$, 30% glycerol. Constructs are named as in **Fig. 1A**. Results are reported as the percentage reduction in biofilm compared to buffer treated biofilms. Data presented represents 4 replicates. Values represent means ± SEM. Asterisks indicate significant differences (single factor ANOVA with t-test post hoc analysis Šidák correction) as compared to 2 (LysK) at that concentration. Adjusted alpha $p < 0.026$. PGH constructs: lysostaphin (L), LysK (K), triple fusion K-L, and triple fusion L-K, as named in **Fig. 1A**.

**Supplementary Figure 4. Mupirocin treatment reduces *S. aureus* nasal colonization in rats.** Reduction in nasal colonization observed when rats were challenged on day 0 with $10^7$ CFU *S. aureus* strain ALR and then treated twice per day on days 5, 6, and 7 with 10 µl of 2% mupirocin calcium ointment (Bactroban Nasal) or petrolatum/softisan placebo. Noses from rats were homogenized and cultured quantitatively on day 10. Each point represents the CFU recovered from an individual rat, and the data were compiled from two independent experiments. Data were analyzed by the Mann-Whitney test, and horizontal lines represent median values. Mupirocin reduced colonization ~98% compared to the placebo control.

**Supplementary Figure 5. Turbidity reduction assay of select PGHs against *S. aureus* Newbould 305.** Bacterial cells were resuspended in lysis buffer to an $OD_{600nm}$ of 1.0 and lysed with 1 µM final concentrations of each PGH. The maximum rate of ΔOD of three experiments are represented as an average with error bars representing SEM. Double asterisks indicate significant difference from parental. Mean comparisons were performed with one way ANOVA ($P = 3.3 \times 10^{-5}$) with t test post hoc analysis with Šidák adjusted alpha ($p<0.01$).

**Supplementary Table 1. Minimum inhibitory concentrations of the PGH constructs against staphylococci, including, bovine mastitis strains, *S. aureus*, MRSA, and coagulase negative staphylococci.**

*All constructs are described in **Figure 1A.**

All NRS strains were obtained from the Network on Antimicrobial Resistance in *Staphylococcus aureus* (NARSA) collections (http://www.niaid.nih.gov/labsandresources/resources/dmid/narsa/Pages/default.aspx)

Ciprofloxacin (Cp), Clindamycin (Cc), Erythromycin (E), Gentamicin (Gm), Linezolid (Lz), Oxacillin (Ox), Penicillin (P), Streptomycin (S), Tetracycline (Te), Trimethoprim-sulfamethoxaxole (SXT), Vancomycin (Va).

| | **Strain** | **Resistant** | **Intermediate** | **Minimum Inhibitory Concentration** (median µg/ml) |
|---|---|---|---|---|

| | | | | | L* | K* | K-L* | L-K* |
|---|---|---|---|---|---|---|---|---|
| | *S. aureus* Newman | | | | 0.77 | 47 | 7.0 | 7.8 |
| | *S. aureus* ALR | S | | | 0.77 | 34 | 7.0 | 4.4 |
| **MRSA Strains** | USA 100 (NRS 382) | Cp, Cc, E, Ox, P | | | 1.2 | 96 | 14 | 20 |
| | USA 200 (NRS 383) | Cp, Cc, E, Gm, Ox, P | | | 1.2 | 34 | 5.5 | 5.9 |
| | USA 300 (NRS 384) | E, Ox, P | Cp | | 1.2 | 75 | 11 | 20 |
| | USA 400 (NRS 123) | Ox, P, Te | | | 1.2 | 75 | 7.0 | 16 |
| | USA 500 (NRS 385) | Cp, Cc, E, Gm, Ox, P, Te, SXT | | | 1.2 | 67 | 7.0 | 12 |
| | USA 600 (NRS 22) | Cp, Gm, Ox, P, SXT | Va | | 1.2 | 75 | 7.0 | 6.8 |
| | N315 (NRS 70) | Cc, E, Ox, P | | | 0.68 | 37 | 7.0 | 16 |
| | Sanger 252 (NRS 71) | Cp, Cc, E, Ox, P | | | 0.58 | 29 | 5.5 | 6.8 |
| | NRS 192 | Ox, P | E | | 1.2 | 80 | 11 | 16 |
| | NRS 193 | Ox, P | | | 1.2 | 37 | 7.0 | 12 |
| | NRS 194 | Ox, P | | | 1.2 | 69 | 14 | 12 |
| | NRS 209 | Ox, P | | | 0.97 | 40 | 7.0 | 12 |
| | NRS 271 | Cp, Lz , Ox, P | E | | 0.58 | 44 | 7.0 | 9.8 |
| **Bovine Mastitis** | *S. aureus* Newbould 305 | | | | 12 | 34 | 11 | 12 |
| | *S. chromogenes* MP02 | | | | 0.58 | 16 | 3.5 | 0.37 |
| | *S. epidermidis* MP04 | | | | 6.2 | 8.4 | 3.5 | 4.2 |
| | *S. hyicus* MP01 | | | | 0.29 | 8.4 | 1.8 | 0.37 |
| | *S. simulans* MP03 | | | | 1.5 | 20 | 3.5 | 5.9 |
| | *S. warneri* MP06 | | | | 1.5 | 28 | 5.3 | 1.5 |
| | *S. xylosus* MP05 | | | | 0.39 | 8.4 | 3.5 | 4.0 |

**Supplementary Table 2. Vectors and DNA Primer sequences.**

| Primer | Sequence |
|---|---|
| LysoAA1NdeI F | 5'-ACGTACGTCATATGGCTGCAACACATGAACATTCAGCAC |
| LysoXhoIR | 5'-GCGCTACTCGAGACCACCTGCTTTTCCATATC |
| LysoSalI F | 5'-ATCATCGTCGACGCTGCAACACATGAACATTCAGCAC |
| LysoAD155XhoIR | 5'-GTTTGTCTCGAGACCTGTATTCGG |
| LysoSH3bSalI F | 5'-GCGCATCTCGAGACAGTAACTCCAACGCCG |
| pET21aStyIR | 5'-CGTTTAGAGGCCCCAAGGGGTTATG |
| LysKaa1Sal F | 5'-GATATA GTCGACGCTAAGACTC |
| **Plasmid** | **Protein expressed** |
| p5301 | Lysostaphin-$His_6$ |
| p3514 | LysK-$His_6$ |
| pSB1101 | Triple fusion K-L-$His_6$ |
| pSB1801 | Triple fusion L-K-$His_6$ |

Underline = Restriction enzyme recognition sequence

Supplementary Figure 1.

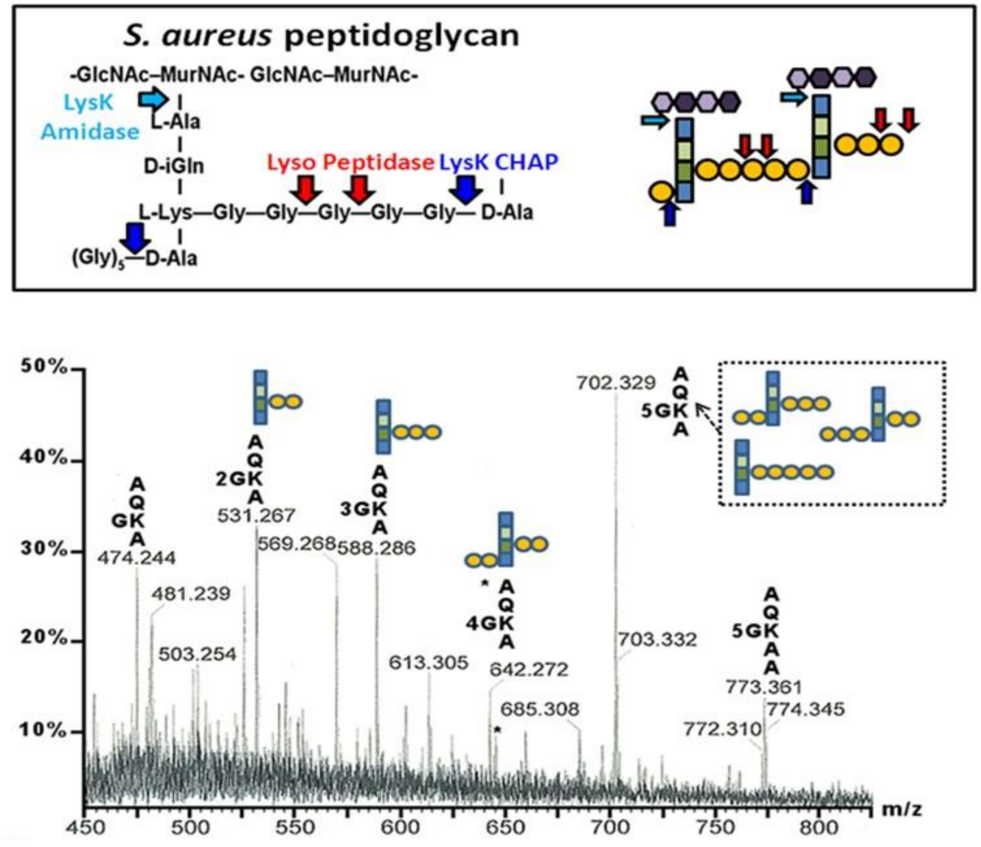


Supplementary Figure 2.

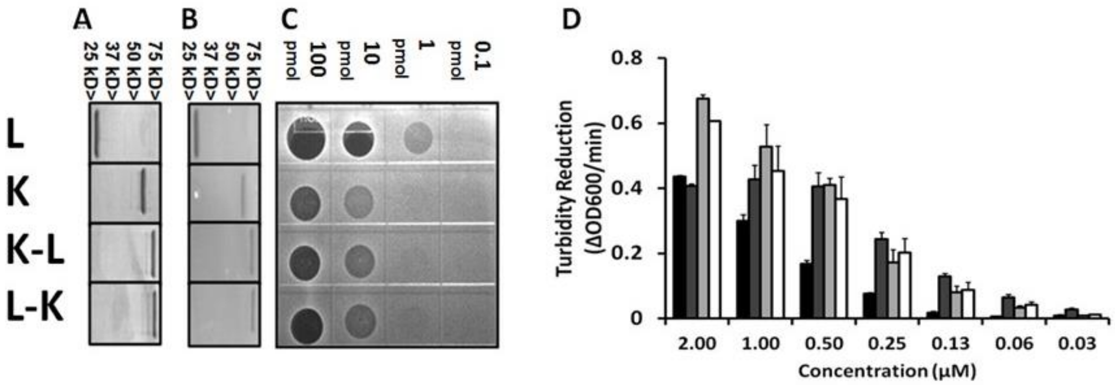

Supplementary Figure 3.

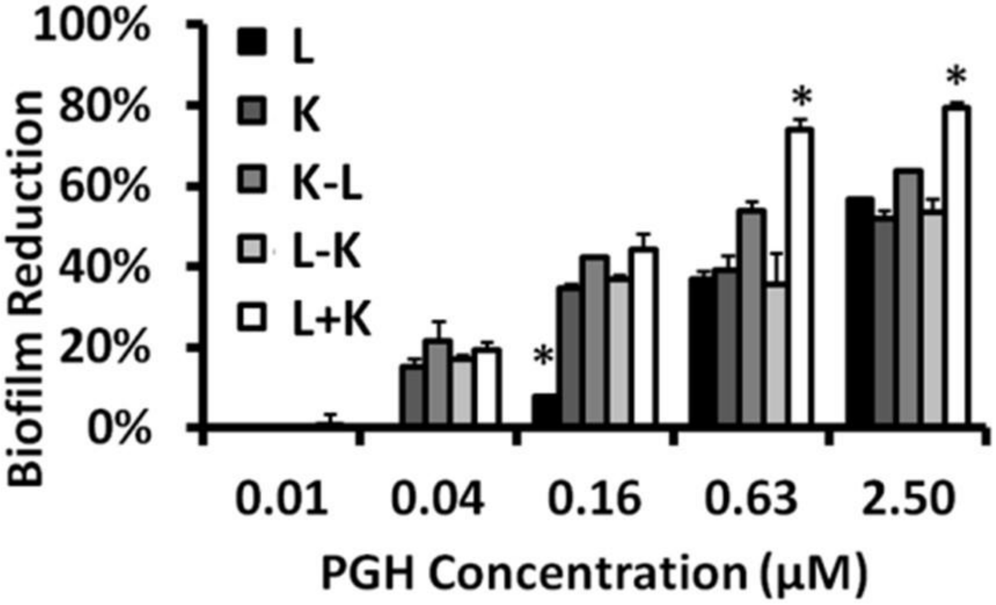


Supplementary Figure 4.

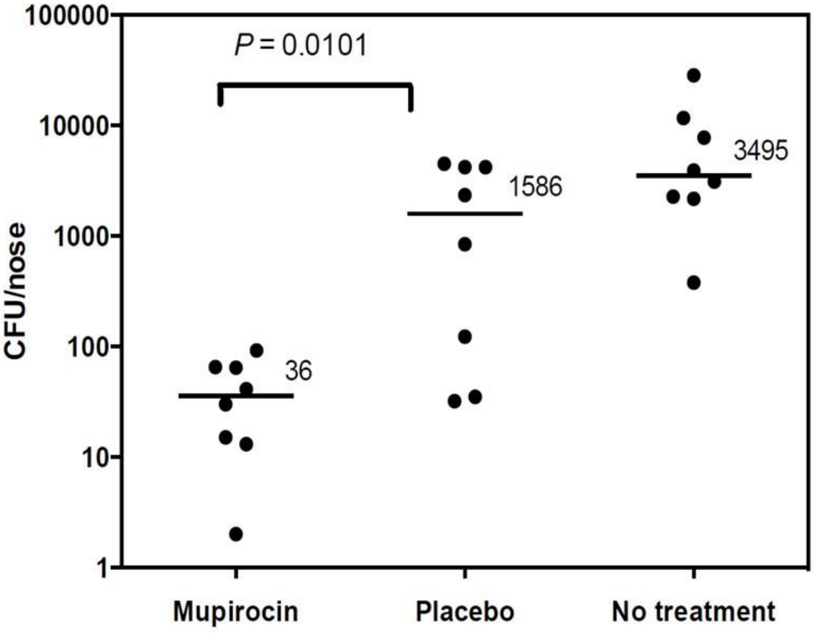

Supplementary Figure 5.

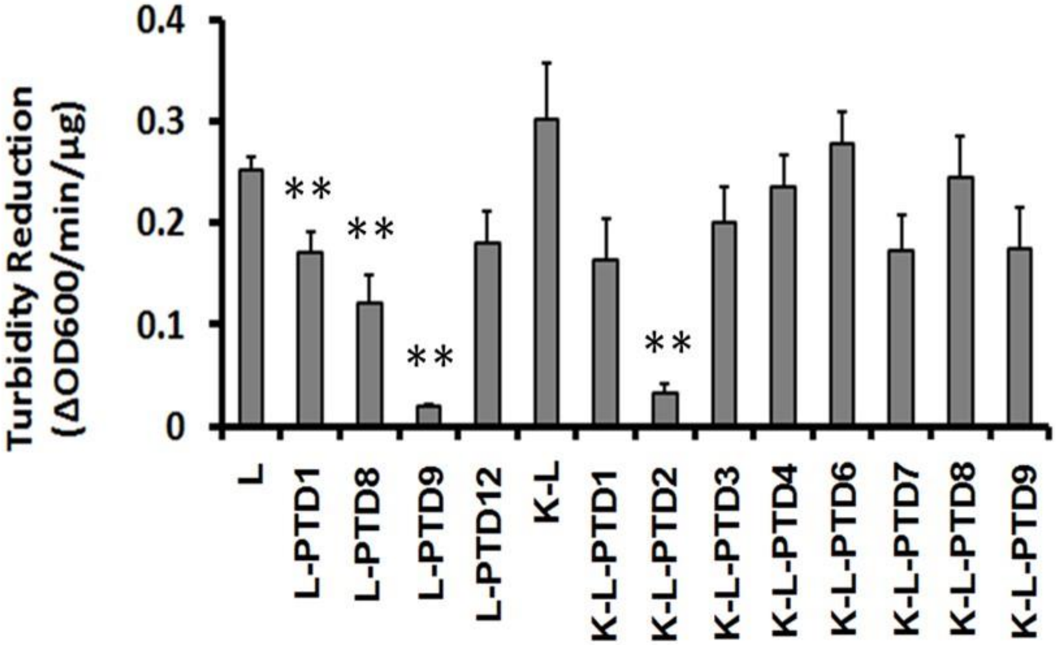

Supplement: Supplementary Information [file srep25063-s1.pdf]
